# Using results from a UK-wide survey to justify choice of comparator for the treatment of severe chronic hand eczema

**DOI:** 10.1186/1745-6215-16-S2-P2

**Published:** 2015-11-16

**Authors:** Isabelle Smith, Sarah Brown, Jane Nixon, Miriam Wittmann

**Affiliations:** 1 Leeds Institute of Clinical Trials Research, Leeds, UK; 2 Leeds Institute of Rheumatic and Musculoskeletal Medicine, Leeds, UK; 3 Centre for Skin Sciences, University of Bradford & Department of Dermatology, Bradford, UK

## 

Treatment of severe chronic hand eczema (CHE) resistant to topical treatment with potent corticosteroids is challenging; in 2013 the Health Technology Assessment Programme released a commissioned call to investigate the most effective treatment for this disease with Alitretinoin specified as the active intervention to evaluate. Existing evidence on systemic treatment options with special focus on Alitretinoin has been summarised in an Evidence Review Group's Report on Alitretinoin for the treatment of severe CHE. The report highlighted that although Alitretinoin has demonstrated efficacy, data are lacking in terms of comparison to other treatment approaches.

The brief required applicants to justify the choice of control intervention; we designed a survey to obtain information on currently used treatment pathways in the UK. A total of 194 UK Dermatologists responded and the results indicated that treatment approaches for severe CHE differ widely among UK dermatologists. PUVA(40.2%) and Alitretinoin(30.9%) were identified as the most frequent first line treatment options for hyperkeratotic CHE, whereas oral steroids(37.6%) were identified as the most commonly used for vesicular CHE, followed by PUVA(26.3%) and Alitretinoin(15.5%). In terms of potential side effects of long term or repeated use, Ciclosporine A(58.2%) and oral steroids(41.2%) were reported to cause most concerns among the surveyed dermatologists. The ALPHA team used the survey results to justify the use of PUVA as the control intervention for first line treatment and to evaluate second line treatments as a secondary objective. The ALPHA trial was approved for funding and is due to open to recruitment in October 2015.

